# Artificial intelligence and machine learning in acute respiratory distress syndrome management: recent advances

**DOI:** 10.3389/fmed.2025.1597556

**Published:** 2025-07-16

**Authors:** Songbei Li, Ruiming Yue, Sen Lu, Jingchao Luo, Xiaoxiao Wu, Zhao Zhang, Mingzong Liu, Yuxin Fan, Yuxuan Zhang, Chun Pan, Xiaobo Huang, Hongli He

**Affiliations:** ^1^School of Medical and Life Sciences, Chengdu University of Traditional Chinese Medicine, Chengdu, China; ^2^Department of Critical Care Medicine of Sichuan Academy of Medical Sciences and Sichuan Provincial People's Hospital, Affiliated Hospital of University of Electronic Science and Technology of China, Chengdu, China

**Keywords:** acute respiratory distress syndrome, artificial intelligence, machine learning, deep learning, reinforcement learning, early prediction, prognostic stratification, phenotype identification

## Abstract

Acute Respiratory Distress Syndrome (ARDS) remains a critical challenge in intensive care, marked by high mortality and significant patient heterogeneity, which limits the effectiveness of conventional supportive therapies. This review highlights the transformative potential of Artificial Intelligence (AI) and Machine Learning (ML) in revolutionizing ARDS management. We explore diverse AI/ML applications, including early prediction and diagnosis using multi-modal data (electronic health records [EHR], imaging, ventilator waveforms), advanced prognostic assessment and risk stratification that outperform traditional scoring systems, and precise identification of ARDS subtypes to guide personalized treatment. Furthermore, we detail AI's role in optimizing mechanical ventilation (e.g., PEEP settings, patient-ventilator asynchrony detection, mechanical power-guided strategies), facilitating Extracorporeal Membrane Oxygenation (ECMO) support decisions, and advancing drug discovery. The review also delves into cutting-edge methodologies such as Graph Neural Networks, Causal Inference, Federated Learning, Self-Supervised Learning, and the emerging paradigm of Large Language Models (LLMs) and agent-based AI, which promise enhanced data integration, privacy-preserving research, and autonomous decision support. Despite challenges in data quality, model generalizability, interpretability, and clinical integration, AI-driven strategies offer unprecedented opportunities for precision medicine, real-time decision support, and ultimately, improved patient outcomes in ARDS.

## 1 Introduction

Acute Respiratory Distress Syndrome (ARDS) is a life-threatening form of respiratory failure characterized by rapid onset, diffuse lung inflammation, and severe hypoxemia. Despite standardized diagnostic criteria (1), ARDS remains challenging to recognize and treat, with mortality rates stubbornly hovering between 35 and 45% in severe cases (1, 2). This grim reality highlights the limitations of current treatment strategies. Over the past few decades, critical care medicine has made significant but limited progress in ARDS management. Lung-protective ventilation strategies, including the use of low tidal volumes, appropriate positive end-expiratory pressure (PEEP) settings, and early prone positioning, have become standard of care (2). These interventions have successfully reduced mortality from the high levels of 65–70% in the 1980s by mitigating ventilator-induced lung injury (VILI) (2). However, behind these achievements lies an undeniable fact: existing therapies are essentially supportive, aiming to create conditions for lung healing rather than directly intervening in the fundamental pathophysiological processes of the disease.

The “ceiling” encountered in current treatment progress is fundamentally due to the profound heterogeneity of ARDS. ARDS is not a single disease but a clinical syndrome triggered by various etiologies (e.g., sepsis, pneumonia, trauma), with vast differences in clinical, imaging, and biological phenotypes (3). This heterogeneity makes “one-size-fits-all” treatment approaches ineffective for all patients, thus forming the current bottleneck in efficacy. Given these challenges, integrating AI and ML into ARDS management holds significant promise for addressing these issues, potentially improving patient outcomes and healthcare efficiency.

The conceptual origins of AI trace back to ancient mythology, where inventors and storytellers imagined the creation of intelligent machines or artificial beings. Centuries later, with the advent of programmable computers, early thinkers immediately questioned the potential intelligence these machines could achieve. Building upon these early ideas, Alan Turing introduced the Turing Test, a conceptual framework to assess whether a machine could convincingly exhibit human-like intelligence, often evaluated through aspects like language comprehension, learning, reasoning, and decision-making. Early AI approaches focused on rule-based systems that replicated human decision-making but encountered limitations when addressing complex tasks. This led to the development of expert systems integrating extensive knowledge bases with reasoning engines. However, these expert systems struggled to effectively handle complex, ambiguous data such as images or natural language. To overcome these limitations, the field shifted toward ML, where algorithms progressively refine their performance through data-driven learning. ML encompasses supervised, unsupervised, and reinforcement learning methods, initially employing straightforward algorithms such as decision trees, logistic regression, and support vector machines, which typically depend on predefined or manually engineered features by human experts. Advances in computational capabilities and data availability have further enabled deep learning, utilizing artificial neural networks to identify intricate patterns in large datasets, significantly advancing fields like image recognition and language processing.

**Figure 1:**
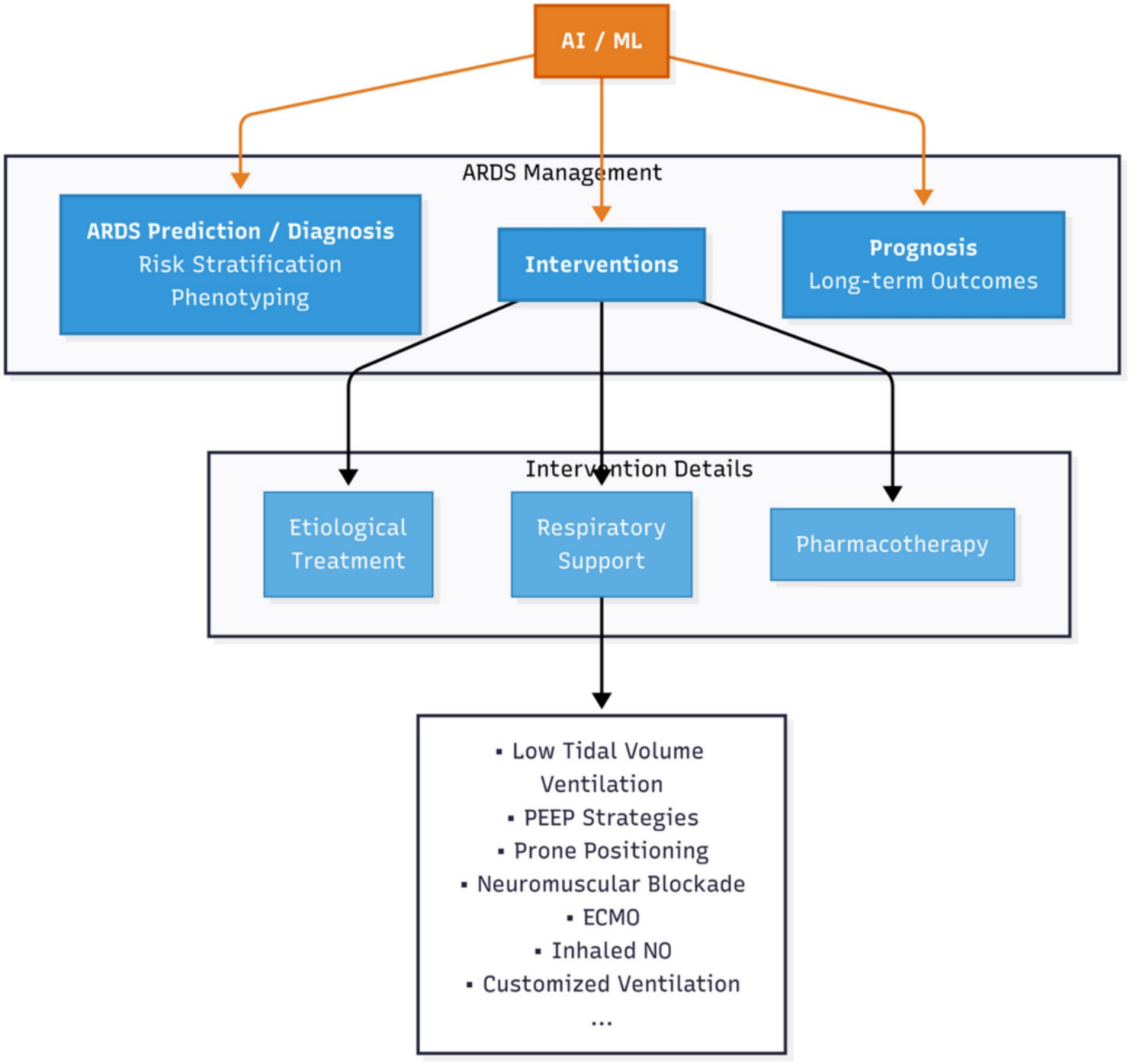
Framework for AI-driven ARDS management. A conceptual model demonstrating the integration of AI and Machine Learning into the management of ARDS. AI/ML informs patient diagnosis and stratification, guides interventions like respiratory support, and aids in predicting clinical outcomes, facilitating a move toward precision medicine.

In healthcare, ML has seen widespread applications, significantly enhancing diagnostic accuracy in medical imaging, improving predictive analytics from electronic health records (EHR), and addressing longstanding healthcare challenges often described as the “impossible trinity”: simultaneously achieving high-quality patient outcomes, cost efficiency, and operational effectiveness. Specifically, in ARDS management, modern ICUs generate vast amounts of data—including high-frequency vital signs, ventilator waveforms, laboratory results, medical imaging, and clinical notes—which exceed clinicians’ ability to assimilate unaided. AI and ML algorithms can analyze and learn complex patterns from these multimodal data sources, offering considerable potential to enhance clinical decision-making by providing more precise and personalized insights into patient diagnosis, prognosis, and treatment. This review aims to provide a comprehensive overview of recent advances in AI and ML for ARDS management. Guided by the framework for AI-driven ARDS management illustrated in Figure 1, we first detail the clinical applications of AI/ML, followed by a discussion of emerging methodologies. Subsequently, we address the practical implementation challenges and shortcomings, and finally, explore promising future directions to further promote personalized care strategies in ARDS.

## 2 AI/ML applications in ARDS management

In ARDS management, AI and ML have become pivotal for enhancing patient outcomes across various clinical domains. This section provides a comprehensive overview of how AI/ML applications are transforming ARDS care, from early detection and diagnosis to personalized treatment strategies. We will explore their utility in predictive analytics, prognostic assessments, and emerging therapeutic guidance, highlighting key advancements and their clinical implications.

### 2.1 Early prediction and diagnosis

**Figure 2:**
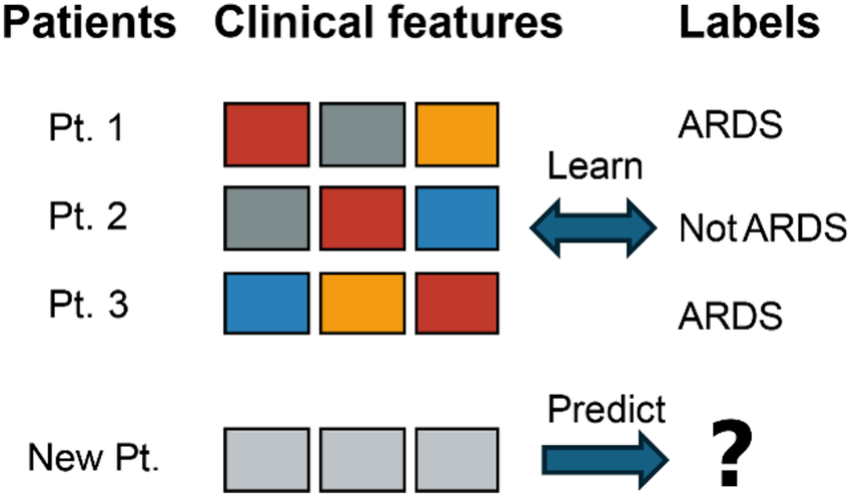
An illustration of a supervised learning workflow for ARDS prediction. Multiple clinical feature sets from the same patient (Pt. 1) are labeled as either “ARDS” or “Not ARDS.” After the model is trained on these labeled examples, it can then predict the label for a new patient’s clinical features.

ARDS is frequently missed or diagnosed late, which significantly hinders timely intervention and worsens patient prognosis (4, 5). The limitations of the Berlin definition have prompted efforts toward a new global definition aimed at increasing diagnostic sensitivity (5). To address this, ML models are being developed to predict ARDS onset hours to days in advance, leveraging diverse data sources such as EHR, medical imaging, and biomarkers (6). See Figure 2 for a schematic overview of the supervised learning workflow used for ARDS prediction. Deep learning, particularly CNNs, demonstrates substantial potential in analyzing CXRs and CT scans for identifying ARDS signs, often surpassing human interpretation accuracy (7). Additionally, VWD has emerged as a novel data source for early ARDS detection through DNNs (8).

Systematic reviews and meta-analyses indicate that ML models generally achieve good performance in ARDS prediction, with pooled AUCs ranging approximately from 0.74 to 0.83, despite persistent heterogeneity and limitations in model quality, including risk of bias, sample size, and validation methods (5). Specific studies highlight this progress: Rehm et al. demonstrated that CNN models utilizing VWD outperformed Random Forest models in ARDS detection (AUC 0.95 vs. 0.88) (8). Sjoding et al. developed DETECT-ARDS, a deep CNN model employing transfer learning, which achieved expert-level accuracy in identifying ARDS signs on CXRs (9). For COVID-19 ARDS, prediction studies integrating clinical data and CT images achieved high AUC values using XGBoost and CNN models (e.g., an integrated model AUC of 0.97) (10).

AI’s capacity to predict ARDS before its full clinical manifestation (e.g., 24–48 h in advance (4)) signifies a paradigm shift from reactive diagnosis to proactive screening and potential early targeted intervention. This predictive capability, if combined with effective early treatments, could fundamentally alter the natural course of ARDS by moving from treating established disease to potentially preventing its full development or mitigating its severity through earlier interventions. An emerging trend is the integration of various data types—including clinical data, imaging, VWD, biomarkers, and unstructured notes analyzed via NLP (4). Models that combine these data sources generally exhibit superior performance, underscoring that a holistic view is crucial for capturing the complex features of ARDS. AI’s strength lies in its ability to synthesize these disparate signals, a task challenging for individual clinicians or simple scoring systems, thus foreshadowing future AI systems as sophisticated data integrators and pattern recognizers (Table 1).

**Table 1:** Application of AI/ML in early prediction and diagnosis of ARDS.

Study (Author, Year)	AI/ML method	Data source & cohort size	Key input features/variables	Key performance metrics (validation type)	Reported progress/novelty	Clinical significance/implications
Rehm et al. (8)	CNN	Single-center ICU (*N* = 100, 50 ARDS, 50 non-ARDS)	VWD	AUC: 0.95 (CNN) vs 0.88 (RF); Acc: 0.84 (CNN) vs 0.80 (RF); Spec: 0.81 (CNN) vs 0.71 (RF) (Internal cross-validation)	Utilized raw VWD for ARDS detection, superior to traditional ML models; found importance of high-frequency information	Suggests VWD as an unbiased early screening tool for ARDS, DL can capture information difficult to extract via manual feature engineering
Sjoding et al. (9)	Deep CNN (121 layers), Transfer Learning	External public CXR (450 k pre-trained), Single-center CXR (8 k fine-tuned), External validation set (another hospital system)	CXR	High accuracy (comparable to physician expert level) (External validation)	Trained ARDS detection model on CXR using transfer learning and large-scale datasets	Can provide rapid diagnostic support, improve ARDS identification rate, ensure timely treatment
Zhou et al. (10)	XGBoost, CNN, Integrated DL model	Single-center (*N* = 103 COVID-19 patients, 23 developed ARDS)	Clinical data (demographics, comorbidities, vital signs, lab tests, etc.), CT images	XGBoost AUC: 0.94; CNN (CT) AUC: 0.96; Integrated model AUC: 0.97 (Internal validation)	Integrated clinical features and CT images with DL model to predict COVID-19 ARDS	Improves COVID-19 ARDS prediction accuracy, aids early identification of high-risk patients
Yang et al. (5)	Various ML algorithms (LR, SVM, RF, DL, etc.)	Pooled data from 17 studies	EHR data, physiological parameters, lab tests	Pooled AUC: 0.7407 (ARDS prediction) (Meta-analysis)	ML shows high efficacy in ARDS prediction, but model quality and external validation need attention	

### 2.2 Prognostic assessment and risk stratification

Despite advancements, ARDS mortality remains high (5). Accurate early risk assessment is critical for guiding treatment intensity, allocating resources effectively, and facilitating communication with patients and their families (11). Traditional scoring systems, such as SOFA, SAPS-II, and APACHE II, have demonstrated limitations in comprehensively assessing ARDS prognosis (4). In response, ML models are increasingly employed to predict mortality in ARDS patients, frequently outperforming these conventional scoring systems by leveraging complex clinical datasets to identify key prognostic factors (4).

A meta-analysis of 21 studies, encompassing 31,291 ARDS patients, revealed that ML models achieved high performance in mortality prediction (pooled C-index of 0.84 for training sets, 0.81 for external validation sets), significantly surpassing the predictive capabilities of SOFA (AUC 0.64) and SAPS-II (AUC 0.70) (11). The application of LSTM models to time-series data derived from APACHE II, SOFA, and SAPS II scores has notably improved AUC values (e.g., APACHE II LSTM AUC 0.898 vs. traditional Logistic Regression 0.777) (12). Furthermore, specific models like Random Forest have also shown robust performance in predicting ARDS mortality (4). Hannon et al. developed a C5.0 ML model that predicted early (7-day) mortality in ARDS patients undergoing prone positioning with an AUROC of 0.78 on test data, utilizing only seven variables (13).

AI models, particularly those that incorporate time-series data such as LSTM models (12), are capable of capturing the dynamic trajectory of a patient’s condition. This provides more nuanced and potentially more accurate prognostic assessments compared to static scores based on worst values within a 24-h period, thereby enabling continuous risk re-evaluation. Given that ARDS is a dynamic condition, AI’s ability to track its evolution and provide updated risk profiles offers a significant advantage over single-snapshot scores. While most studies primarily focus on mortality prediction, there (14) is a growing recognition for the need to predict disability and long-term outcomes (4). This represents an underexplored yet crucial area where AI can provide substantial value in planning rehabilitation and managing patient expectations. Current AI prognostic models are heavily biased toward mortality prediction; however, ARDS survivors often face prolonged and challenging recovery processes. If AI could predict not only mortality but also the likelihood of severe disability or long-term recovery, it could better inform post-ICU care, rehabilitation planning, and more comprehensive discussions about quality of life (Table 2).

**Table 2:** Application of AI/ML in prognostic assessment and risk stratification of ARDS.

Study (Author, Year)	AI/ML method	Data source & cohort size	Key input features/variables	Key performance metrics (validation type)	Reported progress/novelty	Clinical significance/implications
Deng et al. (12)	LSTM	ICU databases (mentions APACHE II, SOFA, SAPS II)	Time-series variables (used to calculate APACHE II, SOFA, SAPS II)	APACHE II LSTM AUC: 0.898 (vs LR 0.777); SOFA LSTM AUC: 0.861 (vs LR 0.715); SAPS II LSTM AUC: 0.897 (vs LR 0.708) (Internal validation)	LSTM optimizes traditional scoring systems, significantly improving prediction accuracy	Emphasizes the importance of temporal dynamic information for prognostic prediction, superior to static scores based on worst values
Hannon et al. (13)	C5.0 Decision Tree (with boosting)	Single-center ICU retrospective data (*N* = 131 ARDS patients undergoing prone positioning)	7 variables: Prone respiratory rate, P/F ratio change, Base excess change, APACHE II, Pre-prone lactate, Sodium change, Bicarbonate change	AUROC (7-day mortality): 0.89 (training set), 0.78 (test set)	Early mortality prediction model for ARDS patients undergoing prone positioning	Aids in identifying non-responders to prone positioning, potentially guiding early consideration of ECMO or alternative treatments
Li et al. (14)	XGBoost (best among 8 ML models); Bayesian optimization; SHAP for interpretability	MIMIC-IV (v3.0), eICU-CRD (v2.0); *N* = 5,732 ARDS patients	54 variables (demographics, vital signs, blood gas, lab tests, comorbidities, severity scores)	Model effectively identifies high-risk ARDS patients (specific metrics not detailed)	Developed interpretable ML mortality risk prediction model	Supports clinical decision-making, promotes early intervention, and improves prognosis
Tan et al. (11)	Various ML algorithms (Systematic Review & Meta-Analysis)	Pooled data from 21 studies; *N* = 31,291 ARDS patients	Complex clinical datasets	Pooled C-index (mortality): 0.84 (training set), 0.81 (external validation set); Superior to SOFA (AUC 0.64) and SAPS-II (AUC 0.70) (Meta-analysis)	ML models outperform traditional scoring tools in ARDS mortality risk assessment	Facilitates early identification of high-risk patients, enabling timely intervention and personalized risk prevention strategies

### 2.3 ARDS subtype/phenotype identification

ARDS is a highly heterogeneous syndrome, contributing to the near-universal failure of many clinical trials due to “one-size-fits-all” approaches (1, 3). Precision medicine aims to identify more homogeneous patient groups through Phenotypes (observable characteristics), Subphenotypes (distinct subgroups with measurable features), and Endotypes (subphenotypes with unique biological mechanisms and predictable treatment responses). The goal is to find “treatable traits” for prognostic enrichment (identifying high-risk patients) and predictive enrichment (identifying treatment responders).

#### 2.3.1 Pioneering discoveries: inflammatory subtypes

A significant breakthrough is the discovery of two major inflammatory subtypes: hypo-inflammatory (P1) and hyper-inflammatory (P2), identified using LCA on large RCT cohorts (15).

The hyper-inflammatory subtype is characterized by elevated inflammatory biomarkers (e.g., IL-6, IL-8, sTNFR-1), more severe organ dysfunction, higher shock rates, and significantly higher mortality (e.g., 90-day mortality of 40–50% vs. 18–26% for P1) (3, 7, 16, 17). The hypo-inflammatory subtype has lower inflammatory markers, less severe disease, and better outcomes (18).

Crucially, these subtypes exhibited markedly different responses to therapies previously deemed ineffective (3, 4, 16, 17, 19). For instance, high PEEP was harmful to hyper-inflammatory but potentially beneficial to hypo-inflammatory patients (3). Fluid management strategies and drugs like simvastatin and corticosteroids also showed differential benefits (3, 4, 16, 17, 19). These findings explain past trial failures, where effects in one subgroup were offset by others (18). The hyper-inflammatory phenotype is consistently found across various ARDS etiologies (sepsis, COVID-19) and even in pediatric ARDS, suggesting it’s a “general critical illness biological feature” (15, 16).

To enable real-time clinical application, supervised ML models, especially GBM, have been developed. These models classify LCA-derived phenotypes using readily available routine clinical data (e.g., demographics, vital signs, lab tests) (7). They show exceptional performance (AUC 0.94–0.95) and are rigorously validated in multiple “real-world” observational cohorts (3, 7). This confirms that complex biological information is encoded in routine data, making bedside phenotyping feasible for precision medicine in the ICU.

Beyond Inflammatory Subtypes, ARDS can be “direct” (pulmonary, e.g., pneumonia) or “indirect” (extrapulmonary, e.g., sepsis) (1). They differ in pathology, imaging, mechanics, and biomarkers (1, 20). Direct ARDS often shows patchy consolidations and reduced lung compliance, while indirect ARDS has diffuse ground-glass opacities and more chest wall compliance issues (1, 20). Despite their clinical relevance, dedicated AI/ML research for classifying these remains a significant gap (9).

#### 2.3.2 Physiological subtypes

AI/ML increasingly derives subtypes from dynamic physiological data:

- “Efficient vs. Restrictive”: ML identified two physiological subtypes based on respiratory mechanics and gas exchange, with “efficient” showing better outcomes (21).
- “Recruitable vs. Non-recruitable”: AI/ML models integrating quantitative CT imaging and respiratory mechanics predict PEEP/recruitment maneuver response for personalized settings (3, 22, 23).
- “Dry, Wet, and Fibrotic”: In severe ECMO-ARDS, LCA identified these three. “Fibrotic” has highest mortality; “Wet” may benefit from higher PEEP (1, 24).

#### 2.3.3 Multi-omics driven endotypes

The frontier involves integrating high-dimensional molecular data (genomics, proteomics) to reveal deep biological mechanisms. AI/ML processes these complex datasets to identify novel biomarkers and therapeutic targets, leading to “metabolic endotypes” for precise drug development (25–27).

The evolution of ARDS subtyping progresses toward a comprehensive, multi-layered, dynamic patient profile integrated by AI. AI/ML is uniquely capable of synthesizing diverse information streams to generate a holistic patient portrait, guiding personalized, combinatorial treatment strategies (Table 3).

**Table 3:** ARDS subtypes identified by AI/ML and their clinical implications.

Subtype category	Specific subtype	Defining characteristics	AI/ML method(s) used	Clinical implications / differential treatment response
Inflammatory (7, 15, 17, 19)	Hypo-inflammatory (P1)	Lower inflammatory biomarkers, less severe organ dysfunction, lower mortality.	LCA (discovery), GBM/XGBoost (classification)	May benefit from liberal fluid strategy; high PEEP potentially beneficial or neutral.
	Hyper-inflammatory (P2)	Elevated inflammatory biomarkers, severe organ dysfunction, shock, higher mortality.	LCA (discovery), GBM/XGBoost (classification)	High PEEP harmful; liberal fluid strategy associated with higher mortality; differential response to statins (e.g., rosuvastatin showed no benefit in this study), potential benefit from corticosteroids.
Physiological (21, 23)	Efficient	Lower mortality, better gas exchange.	Unsupervised ML (GMM), Supervised ML (XGBoost)	Less aggressive ventilation may be sufficient.
	Restrictive	Higher mortality, worse gas exchange.	Unsupervised ML (GMM), Supervised ML (XGBoost)	May require more aggressive lung-protective strategies.
	Recruitable	Lungs open with PEEP/recruitment maneuvers.	LCA/ML (CT imaging & respiratory parameters)	Benefit from higher PEEP and recruitment maneuvers.
	Non-recruitable	Lungs do not open with PEEP/recruitment maneuvers.	LCA/ML (CT imaging & respiratory parameters)	Higher PEEP may be harmful; focus on minimizing VILI.
Dynamic (e.g., ECMO-ARDS) (24)	Dry type	Minimal fluid accumulation.	LCA (discovery)	(Specific treatment implications under investigation)
	Wet type	Significant fluid retention.	LCA (discovery)	May benefit from higher PEEP (e.g., ≥10 cmH₂O) on ECMO.
	Fibrotic type	Evidence of lung fibrosis.	LCA (discovery)	Highest mortality; may require different long-term strategies.
COVID-19 Specific (53)	Dynamic subtypes	Based on longitudinal ventilation parameter trajectories.	Longitudinal LCA	Elevated ventilatory ratio trajectory associated with poor prognosis.
Sepsis-ARDS Specific (4)	Cluster 0 (Mild)	Mildest clinical signs, lowest mortality.	AdaBoost (clustering)	High PEEP may be harmful.
	Cluster 1 (Severe)	Most severe clinical signs, highest mortality.	AdaBoost (clustering)	High PEEP may be harmful.
	Cluster 2 (Moderate)	Moderate severity, longest ICU stay.	AdaBoost (clustering)	High PEEP may reduce mortality.
Multi-omics (25–27)	Metabolic endotypes	Distinct molecular profiles.	AI/ML (high-dimensional data analysis)	Potential for novel, highly targeted drug therapies.

### 2.4 PEEP optimization

Optimizing PEEP selection in ARDS is a critical yet complex endeavor, aiming to enhance oxygenation and prevent atelectrauma while simultaneously avoiding overdistension and hemodynamic compromise (1). Due to significant patient heterogeneity, a “one-size-fits-all” approach to PEEP is insufficient (28). To address this, ML models are being explored to predict physiological responses, such as oxygenation and compliance, at various PEEP levels, thereby facilitating personalized PEEP settings using routinely measured clinical data (29).

Händel et al. developed a multi-task neural network model that utilized MIMIC-III and eICU data to predict PaO2, PaCO2, and Crs approximately 45 min in advance, based on current ventilator settings and patient data. This model demonstrated the ability to simulate the effects of PEEP adjustments, achieving MAPE of approximately 21% for PaO2 and 16% for Crs in the MIMIC-III test set (29). Earlier foundational work, such as that by Chase et al., though not strictly ML, employed model-based methods to estimate patient-specific lung elastance at different PEEP levels to guide optimal PEEP selection (30).

ML models, exemplified by the work of Händel et al. (29), advance beyond traditional trial-and-error or static table methods for PEEP by providing predictive simulations of PEEP effects. This capability allows clinicians to virtually explore PEEP changes and their potential consequences before actual application. Unlike traditional PEEP adjustment, which involves slow incremental changes and observation, the model described in (29) can predict the outcome of PEEP changes, representing a shift from reactive adjustment to proactive, model-guided titration. Its “simulation” capability (29) is a key innovation, offering a foresight into PEEP adjustments. The features identified as important in Händel et al.’s model, such as the last known values of predicted variables and ventilator mode (29), often align with clinical intuition and physiological principles. This alignment can enhance clinician trust and understanding, as AI functions not as a complete “black box” but as a tool that quantifies and predicts known relationships. Clinician trust is a recognized barrier to AI adoption (5); therefore, if an AI model for PEEP selection highlights variables that clinicians already deem important (e.g., current oxygenation, current compliance), its recommendations become more comprehensible and trustworthy. This suggests that AI can augment, rather than replace, clinical reasoning by providing more precise, data-driven insights based on plausible features (Table 4).

**Table 4:** Application of AI/ML in PEEP optimization for ARDS patients.

Study (Author, Year)	AI/ML method	Data source & cohort size	Key input features/variables	Key performance metrics (validation type)	Reported progress/novelty	Clinical significance/implications
Händel et al. (29)	Multi-task Neural Network (NN); Random Forest (RF) for comparison	MIMIC-III (training/testing), eICU (independent testing)	Ventilator settings, vital signs, lab results, and other routine measurements	MAPE (NN, MIMIC-III test set, 30-min blinded prediction): PaO2 21.7%, PaCO2 10.0%, Crs 15.8%. NN outperformed RF on MIMIC-III, but RF was partially superior on eICU	Predicts physiological parameters (PaO2, PaCO2, Crs) approximately 45 min after PEEP adjustment, and can perform simulations	Provides a new method for personalized PEEP titration, without additional cost, aiding clinical decision-making
Chase et al. (30)	Model-based parameter identification (integral method)	Single-center ALI/ARDS patients (*N* = 10)	Airway pressure and flow data	Median absolute percentage fitting error (Edrs): 0.9%; Model-selected PEEP was generally higher than clinically selected values	Identified patient-specific dynamic lung elastance (Edrs) to optimize PEEP	Individualized PEEP selection based on physiological models, aiming for minimal elastance (maximal compliance)

### 2.5 Detection of patient-ventilator asynchrony (PVA)

PVA is a common occurrence in mechanically ventilated patients, with reported incidences as high as 90% in some studies (31). PVA is associated with adverse clinical outcomes, including prolonged ventilation, increased work of breathing, and an elevated risk of barotrauma (31). The visual detection of PVA from ventilator waveforms is inherently challenging and time-consuming for clinicians (31). To address this, AI and ML, including deep learning techniques, are being employed to automatically detect and quantify various types of PVA from ventilator waveforms (flow, pressure, volume) and, in some cases, from Pes (esophageal pressure) (32).

A narrative review by van der Staay et al. identified 13 studies on AI detection of PVA, with 10 reporting sensitivity and specificity greater than 0.9, and 8 reporting accuracy greater than 0.9. Notably, three of these studies focused on ARDS as an indication for mechanical ventilation (32). Another review by Rietveld et al. examined 19 studies, highlighting promising results with average reported sensitivity of 0.80, specificity of 0.93, and accuracy of 0.92. However, they also noted limitations, such as most models being offline, detecting only a small fraction of PVA types (primarily ineffective triggers and double triggers), or lacking external validation (31). Stell et al. developed an ML method for PVA detection that achieved a specificity greater than 90% (33).

While accurate PVA detection is a crucial initial step, its ultimate clinical value lies in linking detection to real-time intervention, such as automatic ventilator adjustments or immediate alerts to clinicians. This “closed-loop” or “decision support” aspect represents the next frontier. Although many studies report high accuracy for PVA detection (31), Rietveld et al. (31) point out that most models are “offline,” meaning historical detection is less useful than real-time identification and actionable insights. The future trend should focus on developing real-time systems that not only detect but also suggest, or even (in highly validated future systems) automatically implement corrective ventilator adjustments. This aligns with discussions in (22) regarding AI adjusting ventilator settings in real-time. The success of AI in PVA detection, often based on raw waveform data, underscores the rich yet underutilized information embedded in these continuous physiological signals. AI excels at discovering complex patterns that may be too subtle or rapid for human clinicians to consistently detect. For instance (33), notes that visual parsing of “whole polysomnograms” is “labor-intensive and error-prone,” and (31) describes visual inspection as “extremely challenging.” AI, particularly deep learning, can process these high-frequency, complex waveform data streams more effectively, as demonstrated by Rehm et al. (8) for detecting ARDS from VWD. This suggests a broader applicability of AI for waveform analysis in other critical care monitoring tasks beyond PVA (Table 5).

**Table 5:** Application of AI/ML in patient-ventilator asynchrony (PVA) detection.

Study (Author, Year)	AI/ML method	Data source & cohort size	Key Input features/variables	Key performance metrics (validation type)	Reported progress/novelty	Clinical significance/implications
Tlimat et al. (32)	Various ML and DL techniques	13 studies, 332 participants, >5.8 million breaths (3 studies for ARDS patients)	Ventilator waveform data (pressure, flow, volume), some with Pes	10/13 models Sens/Spec >0.9; 8/13 models Acc >0.9 (validation methods varied across studies)	AI models show high accuracy across different populations and asynchrony types	AI has great potential in detecting PVA, expected to improve mechanical ventilation management
Rietveld et al. (31)	Rule-based algorithms, ML, DL	19 studies	Ventilator waveforms, some with Pes or EAdi	Average Sens: 0.80, Spec: 0.93, Acc: 0.92 (pooled across studies)	Automated PVA detection techniques are evolving and show promise	Future needs external validation and real-time deployment to optimize personalized ventilation and improve outcomes
Rodriguez et al. (54)	Rule-based algorithm	ARDS patients (invasive ventilation)	Paw and flow waveforms	Pes validation set: Acc: 0.92, Sens ≥0.86, Spec ≥0.91; No Pes validation set: Acc: 0.96, Sens ≥0.74, Spec ≥0.80	Detection of reverse triggering and double triggering	Even without Pes, algorithm can detect specific PVA types with high accuracy

### 2.6 Mechanical power-guided lung protective strategies

VILI remains a significant concern in the treatment of ARDS (34). MP, an emerging concept, integrates variables such as pressure, volume, flow, and respiratory rate to quantify the energy delivered to the lungs and estimate VILI risk (34). High MP values are consistently associated with worse clinical outcomes (34). While it’s lack of the direct use of AI/ML to guide MP-based strategies, ML models have incorporated MP as a key feature for mortality prediction (35), and methods for individualizing MP thresholds using ML are currently being explored (36).

Gattinoni et al. established a foundational formula for MP, indicating that values exceeding 12 J/min are associated with an increased risk of VILI (34). Becher’s simplified formula is commonly used for MP calculation in pressure-controlled ventilation modes (35). Studies have demonstrated that both MP and VR are independently associated with ICU mortality in ARDS patients (35). Chang et al. found that in ARDS patients undergoing PP, post-PP MP/compliance and changes in MP, MP/PBW, and MP/compliance were significantly associated with 28-day mortality, with changes in MP/compliance identified as an independent predictor (HR 7.972) (37). It is important to note that this particular study utilized traditional statistical methods rather than ML for prediction. More recently, Alkhalifah et al. employed various ML models (e.g., LR, RF, SVM, XGBoost) to predict ICU mortality based on MP and other variables, with the XGBoost model demonstrating high prediction accuracy (AUC 0.88). They proposed individualizing mechanical ventilation settings based on real-time physiological variables to reduce predicted mortality (36) (Table 6).

**Table 6:** Application of AI/ML in mechanical power (MP)-guided lung protective strategies.

Study (Author, Year)	AI/ML method	Data source & cohort size	Key input features/variables	Key performance metrics (validation type)	Reported progress/novelty	Clinical significance/implications
Alkhalifah et al. (36)	LR, RF, SVM, Ada boosting, XGBoost, Stacking	Single-center ICU database (ARDS patients)	MP, IBW-normalized MP, VT, RR, ΔP, Ppeak, lactate levels, age, etc.	XGBoost (ICU mortality prediction): Acc: 0.78, Prec: 0.79, Recall: 0.76, AUROC: 0.88 (5-fold cross-validation)	Used ML models to predict ICU mortality and proposed methods for individualizing MV settings based on MP and other covariates	Highlights the potential of individualized MP thresholds to reduce VILI and mortality, promoting data-driven ventilation management
Chang et al. (37)	Cox Regression Model (non-ML)	Multi-center retrospective data (8 hospitals in Taiwan, *N* = 135 ARDS patients undergoing prone positioning)	MP, MP/PBW, MP/compliance (post-prone and changes)	Change in MP/compliance was an independent predictor of 28-day mortality (HR: 7.972, *p* < 0.001)	Found association between post-prone MP-related parameters and ARDS patient mortality	Suggests monitoring post-prone MP parameters can predict prognosis, but needs ML models to further validate its guiding role
Gattinoni et al. (34)	MP calculation formula (non-ML)	-	VT, RR, Ppeak, ΔP, PEEP	MP > 12 J/min associated with increased VILI risk	Proposed MP concept and its calculation method	Provides a basis for quantifying VILI risk, an important input parameter for subsequent ML applications

### 2.7 Prone positioning

PP is a cornerstone therapy for moderate to severe ARDS, significantly improving oxygenation and reducing mortality when applied early and for extended durations (1). However, patient response to PP varies, and predicting who will benefit most remains a challenge (1). ML models are being developed to identify patients most likely to respond positively to PP or to predict mortality in patients undergoing PP. For instance, Hannon et al. developed a C5.0 ML model that predicted 7-day mortality after PP in ARDS patients with an AUROC of 0.78, aiding in the early identification of non-responders (13). Fosset et al. used unsupervised ML to identify three distinct subtypes among ARDS patients undergoing PP, associated with different mortality rates, though their model could not predict individual PP benefit based on existing data (38). This highlights the need for more comprehensive data, such as multi-modal data, to fully capture the complex physiological responses to PP and guide personalized application (Table 7).

**Table 7:** Application of AI/ML in prone positioning for ARDS patients.

Study (Author, Year)	AI/ML method	Data source & cohort size	Key input features/variables	Key performance metrics (validation type)	Reported progress/novelty	Clinical significance/implications
Hannon et al. (13)	C5.0 Decision Tree (with boosting)	Single-center ICU retrospective data (*N* = 131 ARDS patients undergoing prone positioning)	7 variables (see Table 2 in Section 2.2)	AUROC (7-day mortality): 0.78 (test set)	Predicts early mortality risk for ARDS patients undergoing prone positioning	Aids in identifying prone positioning “non-responders,” potentially guiding early consideration of ECMO or adjustment of treatment strategies
Fosset et al. (38)	Unsupervised ML (clustering)	Retrospective observational study (*N* = 353 ARDS patients undergoing prone positioning)	Respiratory mechanics, oxygenation parameters, demographic variables (pre-prone)	Identified 3 subtypes with different 28-day mortality rates (Cluster 3 had highest mortality 56%), but could not predict prone positioning responders	Used unsupervised learning to identify subtypes of ARDS patients undergoing prone positioning and their association with mortality	Suggests heterogeneity in the prone positioning population, but cannot yet guide individualized PP decisions based on this

### 2.8 ECMO support

ECMO is a highly resource-intensive and high-risk rescue therapy for severe ARDS, with its value increasingly recognized, leading to updated clinical guidelines recommending its use for certain severe ARDS patients (39). The challenge lies in precisely identifying patients who will benefit most and optimizing the timing of initiation. ML models are being developed to address these complexities. A significant advancement is “PreEMPT-ECMO,” a hierarchical deep learning model by Zhu et al. that predicts ECMO use up to 96 h in advance using large-scale, multi-modal, time-series data, outperforming traditional ML models (e.g., AUC 0.89 at 48 h in advance) (40). This early warning capability is crucial for patient triage and resource allocation. Furthermore, models like ECMO PAL, a deep neural network trained on a vast international cohort of over 18,000 ECMO patients, has shown superior performance in predicting in-hospital mortality for VA-ECMO patients compared to all existing traditional scoring systems (41). These AI tools provide objective, data-driven predictions to facilitate equitable and efficient allocation of this critical resource, ensuring it is used for patients most likely to benefit (Table 8).

**Table 8:** Application of AI/ML in ECMO support for ARDS patients.

Study (Author, Year)	AI/ML method	Data source & cohort size	Key input features/variables	Key performance metrics (validation type)	Reported progress/novelty	Clinical significance/implications
Zhu et al. (40)	Hierarchical Deep Learning Model (including LSTM)	N3C multi-center database (*N* = 101,400 COVID-19 ICU patients, 1,298 ECMO cases)	Static features (demographics, comorbidities) and multi-granularity time-series features (vital signs, treatments, lab values)	AUROC (ECMO use prediction, 48 h in advance): 0.89; AUPRC: 0.27. Outperformed LR, SVM, RF, XGBoost (multi-timepoint comparison)	Continuously predicts ECMO use risk, up to 96 h in advance; integrates static and dynamic multi-granularity data	Early warning tool, guiding patient triage and ECMO resource allocation, especially during resource scarcity
Stephens et al. (41)	DNN	International cohort (400 + centers, >18,000 ECMO patients)	Pre-ECMO registry variables, (e.g., lactate, age, serum bicarbonate, respiratory rate, endotracheal intubation time, infectious organisms, comorbidities, and other clinical and physiological data)	Outperformed all existing traditional scoring systems in predicting in-hospital mortality for VA-ECMO patients	Breakthrough in predicting ECMO survival with large-scale, generalizable data	Aids in precise patient selection for ECMO, optimizing resource allocation and improving outcomes

### 2.9 Prediction of ventilator weaning and extubation

Successful ventilator weaning and extubation are critical milestones in the recovery of ARDS patients, significantly impacting patient outcomes, ICU length of stay, and healthcare costs. Premature extubation can lead to reintubation and increased mortality, while delayed extubation prolongs ventilation-associated complications. Given the complexity and dynamic nature of ARDS, predicting successful weaning and extubation remains a significant challenge for clinicians. ML models offer a promising approach to leverage vast amounts of physiological and clinical data to provide more accurate and timely predictions, thereby optimizing the extubation process.

However, direct evidence for predicting extubation “success” (as a binary outcome) specifically within pure ARDS cohorts is relatively scarce (42). Most studies on extubation success rates use heterogeneous ICU populations, which may include ARDS patients but do not analyze them as a distinct subgroup (42). These models are typically trained on vast amounts of data from EHRs and large public databases (e.g., MIMIC-III/IV and eICU-CRD) (43). The combination of input features is highly variable, with one review noting studies using anywhere from 8 to 78 variables (42). Common features include patient demographics (e.g., age), disease severity scores (e.g., SAPS II, SOFA), vital signs (e.g., heart rate), laboratory results (e.g., BUN, PaO2), neurological status (e.g., Glasgow Coma Scale), and ventilator parameters (e.g., MV duration, PEEP, FiO2) (42). A significant trend is the utilization of high-frequency time-series data from ventilators, which provides a rich and continuous view of the patient’s respiratory status (44).

The ultimate goal of AI/ML applications is to improve patient outcomes. Several studies have shown that integrating AI prediction models into clinical weaning protocols can yield tangible benefits. One notable study demonstrated that AI-assisted protocols significantly reduced average MV duration, ICU length of stay (LOS), and total hospital stay compared to control groups without AI (45). Another study reported a 0.5-day reduction in average ventilation days required for successful weaning after AI intervention (46). These findings suggest that AI can serve as a practical tool to help clinicians make more timely and accurate weaning decisions, thereby improving healthcare quality and resource utilization efficiency (47). This is particularly crucial for ARDS patients, where the unique pathophysiological challenges necessitate highly precise and individualized weaning strategies. Future research should focus on developing and validating AI models specifically tailored to predict extubation success in pure ARDS cohorts, leveraging their unique physiological characteristics to bridge this critical knowledge gap.

### 2.10 AI-driven drug discovery

Perhaps the most transformative potential contribution of AI to ARDS treatment is its ability to shift therapeutic strategies from purely supportive care to targeted molecular interventions addressing the disease’s core biological mechanisms. Generative AI is revolutionizing drug discovery (48).

The discovery of Rentosertib serves as an excellent example. This is the first drug whose biological target (TNIK) and the drug molecule itself were discovered by generative AI37. The process involves AI biology engines like PandaOmics analyzing vast biological data (e.g., genomics, proteomics) to identify novel disease-related drug targets. Subsequently, generative chemistry engines like Chemistry42 design and optimize new small molecule compounds with desired pharmacological properties for the identified targets (48). This entire process is significantly faster than traditional methods and has shown higher success rates in early clinical trials (48).

Although IPF, its targeted fibrotic pathway is highly relevant to the fibroproliferative phase many ARDS patients enter during prolonged illness. Therefore, the success of this method in related lung diseases provides strong conceptual validation for its direct application in ARDS research. In the future, AI can analyze multi-omics data from ARDS patients to discover novel anti-inflammatory or anti-fibrotic drug targets (49), potentially leading to the development of the first truly specific ARDS therapies.

For the past half-century, the battle against ARDS has primarily focused on supportive care to mitigate VILI. AI-driven drug discovery opens a crucial second front in this battle: directly combating the core biological processes driving the syndrome. This represents the greatest potential for a step-change reduction in mortality. If AI can help develop a drug that effectively blocks the inflammatory storm or prevents lung fibrosis, its impact would far exceed any optimization of PEEP or prone positioning. This directly addresses the fundamental question of AI’s role in “significantly reducing mortality.”

## 3 Emerging AI/ML techniques in ARDS research

Building upon the established applications of AI/ML in ARDS management discussed previously, the field of AI is continually evolving. Several cutting-edge machine learning techniques, including the transformative rise of LLMs and their agentic applications, hold particular promise for further advancing ARDS research and care. These methods, some only recently applied in medicine, can address current limitations by modeling complex relationships, leveraging diverse data, and enabling collaborative training. We highlight a few notable ones:

### 3.1 Graph neural networks (GNNs)

Traditional deep learning (like CNNs, RNNs) handles data in Euclidean formats (grids of pixels, sequences of time points). But much of healthcare data is relational – patients connected to clinical events, or physiological variables interacting in networks. GNNs are a class of models that operate on graph-structured data, learning representations for nodes (e.g., patients or clinical variables) by aggregating information from their neighbors. In critical care, one can construct graphs such as a patient-similarity network (nodes are patients, edges connect patients with similar profiles), or knowledge graphs linking clinical concepts. GNNs can capture the interdependencies in such graphs. A recent survey identified a surge of interest in GNNs for clinical risk prediction using EHRs (49). Over 5 studies since 2020 have explored GNN architectures (especially Graph Attention Networks) on ICU datasets like MIMIC-III to predict outcomes or diagnoses (49). For ARDS, one might imagine a GNN that links patients by common risk factors and learns a representation of “ARDS propensity” that could improve prediction accuracy, or a GNN that models relationships between different organ systems’ dysfunctions to better predict ARDS onset in sepsis. Early work shows GNN-based models can outperform flatter models by utilizing relationship data – for example, connecting current patients to past patients who had similar trajectories can help forecast deterioration. While GNNs have not yet been widely applied specifically to ARDS, the technique’s ability to naturaly incorporate heterogeneous data and relationships (such as a graph of ventilator settings changes connected over time, or molecular interaction networks in ARDS pathology) makes it a promising avenue for future research.

### 3.2 Multi-modal learning

ARDS diagnosis and management rely on integrating data from multiple sources (clinical measurements, imaging, lab tests, waveforms, etc.). Multi-modal ML refers to algorithms that jointly learn from different data types. We already discussed examples like combining CT scans with EHR data for ARDS prediction (4). Moving forward, more sophisticated multi-modal architectures (such as models that fuse time-series vitals, lab trends, CXR images, and even genomics) could provide a comprehensive “holistic” prediction. Techniques like cross-modal attention allow a model to focus on relevant features in one modality based on patterns in another – e.g. an algorithm might learn that worsening oxygenation (vitals modality) together with new bilateral opacities (image modality) is a stronger ARDS signal than either alone. Multi-modal deep learning has been greatly enabled by the increasing availability of synchronized datasets (for example, MIMIC-IV linking ICU data with radiology images). For ARDS, multi-modal models have shown outstanding performance: one model combining clinical data + CXR features achieved 0.95–0.97 AUROC for ARDS vs. non-ARDS in a COVID cohort (6). As these techniques mature, we expect AI “ensembles” that mirror the way clinicians synthesize labs, imaging, and exam findings. This could also facilitate continuous monitoring – e.g. a system that continuously ingests ventilator waveforms, radiographs, and labs to update the probability of ARDS or to detect transitions (onset of fibroproliferative phase, etc.) and suggest timely interventions.

### 3.3 Causal inference and counterfactual prediction

A common critique of standard ML is that it’s correlation-based – it might predict outcomes, but it does not explain what will change the outcome. Causal inference techniques aim to estimate cause-effect relationships from data. We already noted how causal ML (like Bayesian causal forests) can identify heterogeneous treatment effects in ARDS (50). Another aspect is *counterfactual prediction*: predicting what would happen under different hypothetical treatments. For example, “would this patient’s oxygenation improve if we increase PEEP by 5?” This typically requires causal modeling or causal assumptions. New algorithms such as causal reinforcement learning or deep counterfactual networks attempt to learn these causal relations from observational data. In ARDS, where RCTs are difficult for every possible intervention, such methods can *inform treatment decisions by simulating interventions* in silico. A concrete emerging method is “*target trial emulation*” combined with ML – structuring observational ICU data to mimic a randomized trial and then using ML to adjust for confounders and estimate an intervention’s effect. One study applied this to proning and steroid use in ARDS, using causal forests to suggest that certain subgroups derive more benefit from these therapies (findings that align with clinical intuition, but achieved with computational analysis) (3, 38). Causal inference is still a developing field in ML, but as it progresses, clinicians may gain AI tools that not only risk-stratify but also answer “what if” questions for ARDS management, providing a form of evidence-based guidance derived from big data.

### 3.4 Federated learning

A major barrier in developing robust ARDS AI models is data availability and privacy. ARDS patients are relatively rare at a single center, and data are siloed across hospitals. FL is an approach that enables AI model training on decentralized data – data stay at each institution, and only model updates (gradients) are shared to build a global model, preserving patient privacy. This is extremely pertinent for critical care: an ICU network could collaboratively train an ARDS prediction model on tens of thousands of patients without ever exchanging raw health data. Studies have shown that federated models can achieve performance on par with traditional centrally-trained models. For instance, a 2022 investigation into ICU mortality prediction found that a federated deep learning model performed equally as well as a model trained on pooled data, and significantly better than models trained on individual hospitals alone (11). This indicates FL can harness the “wisdom” of multiple institutions to improve generalizability. In the ARDS context, one can envision a federated effort where many centers contribute to training a robust ARDS early-warning model. Each ICU’s data (demographics, ventilator readings, etc.) helps the model learn, but patient privacy is maintained. Such a model would likely generalize better to new hospitals (a common issue where models fail when applied to external sites) because FL inherently brings heterogeneity during training. Federated learning frameworks also naturally address data ownership and legal concerns, making multi-center AI feasible.

While FL addresses data silos and privacy, variations in clinical practices across institutions (e.g., patient populations, protocols, data collection) can significantly impact model performance and generalizability. Such heterogeneity can lead to reduced accuracy, perpetuated biases, or “domain shift” when models are applied to new environments. To mitigate this, future efforts should focus on robust aggregation strategies, domain adaptation techniques, and standardizing data collection to ensure more generalizable and reliable federated models, along with transparent reporting of dataset characteristics and external validation.

### 3.5 Self-supervised learning

Supervised learning has so far been the workhorse of medical AI, successfully powering tools for prediction and prognosis in ARDS, yet it remains constrained by its reliance on the scarce labeled data. High-quality labels (e.g., expert adjudicated ARDS diagnosis or outcomes) are expensive and time-consuming to obtain. SSL offers a way to pre-train models on *unlabeled data* to learn useful representations, which can then be fine-tuned on smaller labeled datasets. In ICU data, SSL methods have been applied to physiological time-series and clinical notes. For example, transformer-based models have been self-supervised on large corpora of adult ICU data by masking parts of the data and teaching the model to predict them (a “fill in the blanks” pre-training). The resulting model captures latent structure in the patient data. Remarkably, one study showed that a self-supervised model trained on adult ICU stays could be fine-tuned to predict pediatric patient outcomes with high accuracy – in fact, its performance was non-inferior to a logistic regression trained on the pediatric data directly (8). Specifically, the SSL-pretrained model achieved an average AUROC of 0.90 on various pediatric outcome prediction tasks, versus 0.87 for the traditional model (8). This demonstrates SSL’s power to *transfer knowledge* from large adult datasets to smaller pediatric (or other domain) datasets, effectively circumventing limited labels. In ARDS, SSL could be used to learn general cardiorespiratory dynamics from hundreds of thousands of ICU stays (most of which will not develop ARDS) and then adapt that knowledge to identify subtle precursors of ARDS with relatively few positive cases for training. Similarly, self-supervised vision models could learn from the abundance of chest X-rays without needing each to be labeled as “ARDS” or not – by learning to represent normal vs. abnormal lung patterns – and then detect ARDS-specific patterns with minimal supervised tuning. As SSL techniques (such as contrastive learning, masked modeling, and transformer pre-training) evolve, we expect their adoption in ARDS research to grow, enabling more robust models especially in low-data settings. Future ARDS studies might use foundation models (pretrained on general ICU data or even multimodal medical data) and achieve strong performance with only a handful of ARDS-specific labels, which is extremely useful for rare syndromes.

### 3.6 Large language models (LLMs) and agent-based AI

The advent of LLMs and the subsequent development of LLM-based agents represent a profound paradigm shift in AI, moving from passive prediction to autonomous action and complex problem-solving. These models, particularly those built on the Transformer architecture, are pre-trained on vast text corpora, endowing them with unprecedented capabilities in natural language understanding, generation, and reasoning.

#### 3.6.1 LLMs as intelligent callers and interpreters for specialized models

Traditional supervised learning models (such as RF, SVM, etc., mentioned above) perform well in clinical prediction tasks, but their application presents some inherent challenges. They typically require large, high-quality datasets annotated by experts for training, and rely on complex manual feature engineering to extract meaningful variables, a process that is both time-consuming and requires deep domain knowledge (41). The advent of LLMs offers a new possibility for clinical prediction. Their core advantage lies in their powerful zero-shot or few-shot learning capabilities, meaning they can perform tasks with no or very few labeled samples (5). Because LLMs have encoded rich clinical knowledge in their massive pre-training data (including a large volume of medical literature and text), they can directly process raw text, eliminating the tedious feature engineering step (3). However, current research indicates a clear trade-off in performance. For clinical prediction tasks based on structured data, traditional ML models trained on local data (such as gradient boosting trees) still significantly outperform general LLMs (such as GPT-4) that have not undergone specific fine-tuning, in key metrics such as AUC and model calibration (27). This means that for purely predictive tasks, specialized models remains the superior choice. When LLMs are provided with a small number of high-quality examples (few-shot examples) and employ carefully designed prompting strategies, the performance gap between them and traditional ML models significantly narrows (27). This reveals a key potential role for LLMs in the clinical AI ecosystem: they may not replace highly optimized specialized predictive models, but rather serve as intelligent “callers” and “interpreters” for these models. The flexibility of LLMs and their powerful ability to process unstructured information make them an ideal “brain” that can use specialized predictive models as “tools” within a larger agent-based framework, thereby complementing the strengths of both.

#### 3.6.2 LLMs for unstructured data integration and enrichment

Additionally, LLMs can empower specialized predictive models by transforming how unstructured clinical data—a significant portion of EHRs—can be leveraged. Unlike early NLP techniques limited by keyword-based approaches or extensive feature engineering, LLMs can directly process raw text from clinical notes, radiology reports, and discharge summaries. This allows them to extract nuanced information and contextual clues vital for accurate diagnosis and risk assessment in ARDS. For instance, LLMs can summarize daily ICU progress notes, identify potential risks, and even translate natural language queries into structured SQL commands for database interaction [e.g., ICU-GPT (31)]. This capability significantly enhances the richness of data available for ARDS models, moving beyond structured vital signs and lab results to incorporate the full narrative of a patient’s journey. By providing a powerful means to integrate and interpret the complex, unstructured narrative of a patient’s clinical course, LLMs enrich the overall data landscape, thereby empowering specialized predictive models for ARDS management.

#### 3.6.3 Multimodal large language models (M-LLMs)

The next frontier involves M-LLMs, which integrate and process information from diverse sources beyond text, including medical images (CXR, CT, ultrasound), physiological waveforms (ventilator data), and even genomic data. In ARDS, M-LLMs can fuse these heterogeneous data streams to provide a holistic patient assessment. For example, an M-LLM could simultaneously analyze a patient’s chest CT scan, read the radiologist’s report, interpret real-time ventilator waveforms, and understand the clinician’s notes to generate a dynamic ARDS risk score or suggest personalized treatment adjustments. This integrated approach mirrors the complex decision-making process of human clinicians, offering a more comprehensive understanding of the patient’s evolving condition.

#### 3.6.4 LLM agents: from prediction to action

LLM agents extend the capabilities of static LLMs by enabling them to plan, act, and reflect autonomously to achieve complex goals. Unlike traditional LLMs that generate single-turn responses, agents can break down high-level clinical problems into sub-tasks, interact with external tools (e.g., specialized image analysis models, drug databases, clinical guidelines), and iterate through a “perceive-think-act” loop. In ARDS management, an LLM agent could automate diagnosis and subtyping by calling specialized AI tools for image analysis or biomarker clustering, rapidly identifying ARDS and its specific phenotypes (e.g., hyper-inflammatory vs. hypo-inflammatory), which is crucial for personalized treatment. It could also guide personalized ventilation, building on reinforcement learning, where a “ventilator agent” continuously analyzes dynamic physiological data (lung mechanics, gas exchange) and suggests optimal PEEP, tidal volume, or respiratory rate settings, while minimizing VILI risk, operating in a “human-in-the-loop” fashion and providing explainable recommendations for clinician review. Furthermore, an agent could assist in drug therapy by identifying the most appropriate immunomodulatory drugs for a specific ARDS phenotype, cross-referencing guidelines and patient contraindications, and even drafting medication orders.

The development of multi-agent systems, where different LLM agents collaborate (e.g., “predictor” and “critic” agents in EHR-CoAgent (8), or multi-disciplinary team simulations in MedAgents (39)), further enhances the robustness and reliability of AI in critical care. These systems can simulate complex clinical reasoning, identify biases, and self-correct, paving the way for more sophisticated decision support.

### 3.7 Reinforcement learning (RL)

RL is a powerful machine learning paradigm that addresses complex sequential decision-making problems in critical care. Its core premise is modeling a process where an “agent” (the AI) interacts with an “environment” (the patient) to maximize cumulative rewards. In the ICU, the AI agent recommends treatment plans, and the patient’s physiological state evolves in response to these interventions. This interaction is formalized through a Markov Decision Process (MDP), which defines the patient’s state, available actions, and the clinical objectives translated into a reward signal (39).

**Figure 3:**
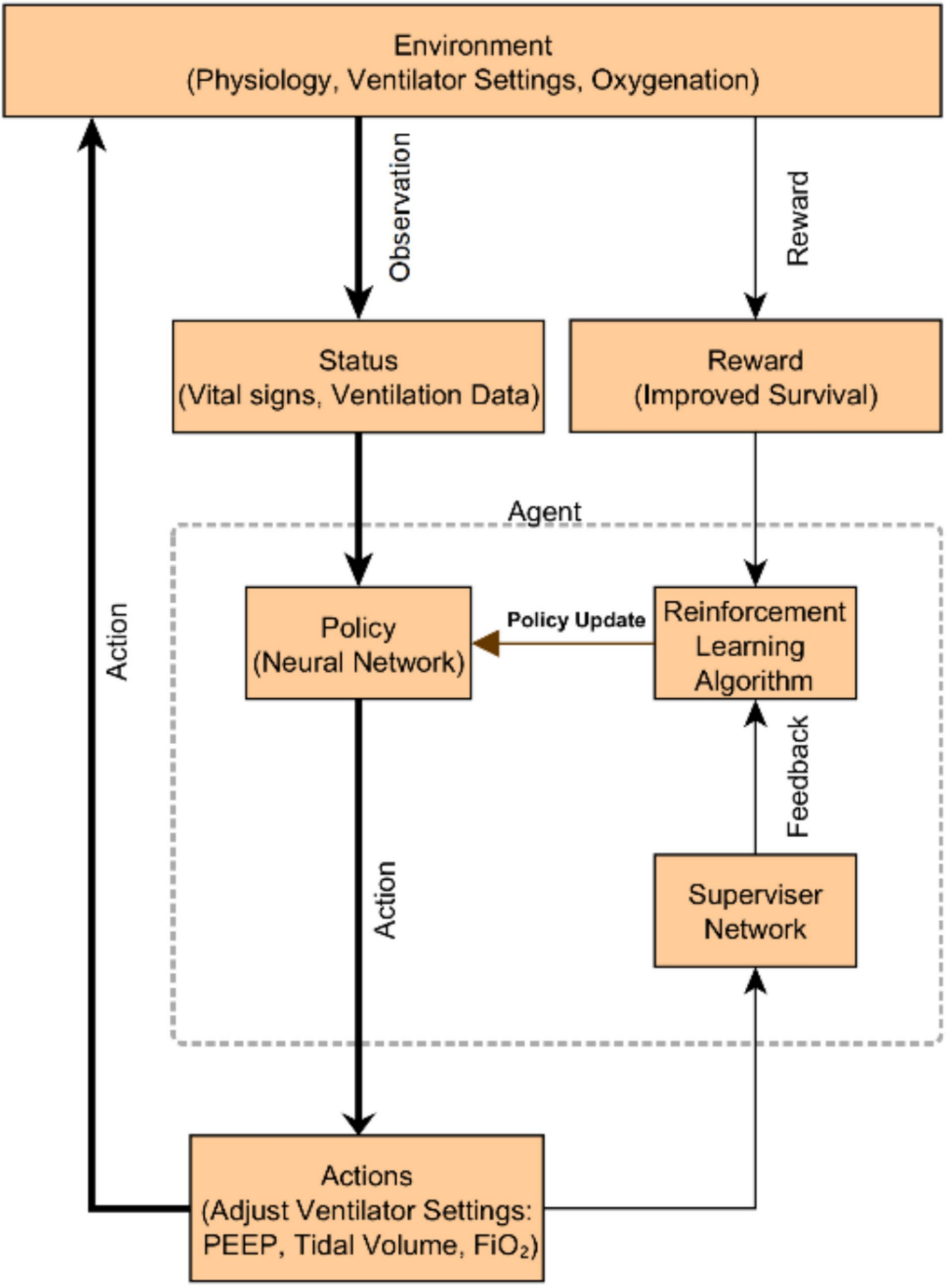
A schematic overview of the reinforcement learning–based ventilation system. The environment encompasses patient physiology, ventilator settings, and oxygenation. The agent observes the patient’s status (vital signs and ventilation data), generates actions (adjusting PEEP, tidal volume, FiO₂), and receives rewards for improved survival. A supervisor network provides safety feedback to the reinforcement learning algorithm, which continuously updates the neural network policy to optimize ventilator management. Such an AI-driven decision support system aims to personalize ventilation in ARDS, improving outcomes.

One prominent example is using RL to optimize mechanical ventilation. In an RL framework, an AI “agent” can learn to adjust ventilator settings in response to a patient’s state, with the goal of minimizing long-term harm (e.g., VILI) and improving survival. Recently, Liu et al. developed an RL-based decision support called “EZ-Vent” to recommend personalized vent settings for ICU patients on mechanical ventilation (10). They trained a policy network on two large critical care databases (MIMIC and eICU) with >26,000 combined ventilated cases, using an off-policy deep Q-learning algorithm. The agent’s action space included suggestions for higher or lower PEEP, tidal volume, and FiO₂ levels depending on patient conditions. Off-policy evaluation (a retrospective simulation on held-out patient trajectories) showed that the RL policy would have achieved better patient outcomes than the observed clinician decisions (10). Specifically, the AI-recommended ventilation strategies were associated with an estimated reduction in hospital mortality (e.g., in one cohort, 12.1% mortality under the AI policy vs. a higher actual mortality in similar patients) and more time with optimal oxygenation and blood pressure ranges (10). These improvements came without increasing adverse events, aside from more frequent ventilator setting adjustments as the AI fine-tuned therapy (51). Such findings highlight the potential of reinforcement learning: the agent can discover an optimal policy by trial-and-error on data, balancing complex trade-offs (oxygenation vs. ventilation pressures) that challenge human intuition. Figure 3 illustrates an example framework of using an RL agent (“policy network”) supervised by clinical data to iteratively improve ventilator settings. Notably, while these results are retrospective, a prospective clinical trial (the DeVENT study) recently tested a decision support system based on a patient-specific physiological model (“digital twin”) to advise on PEEP adjustments in ARDS (51). The trial reported improved lung physiology (oxygenation index, ventilatory ratio) in the intervention arm, although no significant difference in the primary outcome (driving pressure) (51). Importantly, clinicians adhered to about 60% of the AI’s recommendations, and even this partial adoption yielded measurable benefits in gas exchange (51). This points to both the promise and current limitations: AI can provide reasonable treatment advice, but integrating it into practice and demonstrating hard outcomes benefits will require further refinement and clinician acceptance.

Beyond traditional RL, the emergence of LLMs is driving a new trend: LLM-based RL for clinical reasoning. While conventional RL handles structured data, LLMs can process complex, unstructured text from clinical notes and reports, which contain rich medical information (36). This allows LLM-based RL to move beyond simple optimization to perform sophisticated reasoning tasks, such as generating hypotheses, interpreting multimodal data, and engaging in diagnostic processes that mimic human clinicians. These advanced architectures, like EHRMind and Hypothesis-Driven Diagnosis (LA-CDM), aim to not only recommend actions but also explain the “why” behind them, addressing the “black box” problem of AI and fostering greater clinical trust. They learn from human feedback and verifiable outcomes, suggesting a future where AI acts as a “reasoning co-pilot” that continuously refines its strategies through human-in-the-loop interaction (36, 39, 41).

## 4 Challenges and future directions

Despite the significant advancements and promising applications of AI and ML in ARDS management, several challenges must be addressed to ensure their successful and widespread clinical integration.

One fundamental challenge lies in the distinction between *algorithmic performance* and *clinical impact*. Many research reports focus on algorithmic performance metrics on retrospective datasets, such as AUC-ROC (52), but this does not guarantee their utility in the real clinical world. Here lies a fundamental difference: algorithmic performance does not equal clinical impact. A model might achieve 99% prediction accuracy on a test set, but if it errs at critical decision points, or if its alerts lead to disruption of clinical workflow and “alert fatigue,” then it may be clinically useless, or even harmful. For example, a model used to predict ICU readmission risk, even if its AUC-ROC is high, must answer more important questions: Do clinicians trust its alerts? Do interventions triggered by alerts truly prevent patient readmission, or do they lead to unintended consequences such as unnecessary prolonged hospital stays? Therefore, the field must shift from computer science-centric evaluation (“How accurate is the model in predicting X?”) to clinical trial-centric evaluation (“Does using this model to predict X truly improve patient outcomes?”). The stepped-wedge cluster randomized controlled design of the ASIC trial is a prime example, evaluating endpoints not based on algorithmic accuracy, but on clinically critical key performance indicators such as ARDS diagnosis rate, guideline adherence, days of organ dysfunction, and mortality (7). This is a higher hurdle that AI tools must cross to prove their true value.

Other key challenges include data quality and availability, as AI models heavily rely on large, high-quality, and diverse datasets. In ARDS, data can be fragmented, incomplete, or inconsistent across different institutions, and the lack of standardized data collection protocols and interoperability between EHR systems hinders the development of robust and generalizable models. Model generalizability and external validation are also significant concerns, as models developed at a single center often perform poorly when applied to external populations due to differences in patient demographics, clinical practices, and data characteristics; rigorous external validation across diverse settings is crucial but often lacking. Furthermore, interpretability and explainability pose a challenge, as many advanced AI models, particularly deep learning models, operate as “black boxes,” making it difficult for clinicians to understand how decisions are made, which can reduce trust and hinder clinical adoption, especially in critical care settings where accountability is paramount. Clinical workflow integration is another significant hurdle, as seamlessly integrating AI tools into existing clinical workflows without disrupting patient care or increasing clinician burden requires user-friendly interfaces and efficient alert systems. Ethical and legal considerations, such as patient privacy, data security, algorithmic bias, and accountability for AI-driven decisions, also require careful consideration and robust regulatory frameworks. Finally, alert fatigue, caused by over-alerting from AI systems, can lead to clinicians ignoring important warnings, thus balancing sensitivity and specificity to provide actionable and relevant alerts is critical.

Despite these challenges, the trajectory of AI in ARDS management is exceptionally promising, poised to redefine critical care. Key future directions include *digital twin patient simulations*, enabling clinicians to virtually test treatment strategies on a continuously updated, personalized patient model before real-world application. *Real-time AI-driven decision support systems* will move beyond retrospective analysis, offering dynamic, bedside recommendations for ventilator settings, fluid management, or drug titration based on continuous patient data. The *integration of multi-omics data* (genomic, proteomic, metabolomic) promises a deeper understanding of ARDS pathophysiology, leading to novel biomarkers and highly targeted therapies. Furthermore, *reinforcement learning* will empower AI agents to learn optimal, adaptive treatment policies from patient data, continuously refining interventions in real-time. Crucially, *human-in-the-loop AI* will ensure these advanced systems augment, rather than replace, human clinicians, fostering trust and leveraging the synergistic strengths of AI and human expertise. These innovations collectively pave the way for truly personalized, proactive, and ultimately life-saving ARDS care.

## 5 Conclusion

ARDS continues to be a major challenge in critical care, marked by high mortality and profound heterogeneity. The integration of AI and ML offers a transformative approach to address these complexities, ushering in a new era of precision medicine for ARDS management. This review has demonstrated how AI/ML models excel in synthesizing vast, multi-modal datasets to provide timely, accurate, and nuanced insights across the entire spectrum of ARDS care.

Key advancements highlighted include highly accurate predictive models for ARDS onset and mortality (5, 11), sophisticated classifiers for identifying distinct ARDS phenotypes (7), and innovative strategies for optimizing ventilator settings such as PEEP and mechanical power. Emerging techniques like Graph Neural Networks, Multi-Modal Learning, Causal Inference, Federated Learning, Self-Supervised Learning, and the burgeoning field of LLMs with agentic capabilities are poised to further revolutionize the field. These methods promise more robust data integration, privacy-preserving collaborative research, and increasingly autonomous decision support, moving beyond passive prediction to active intervention.

Despite the significant progress, substantial challenges remain, particularly concerning data quality, model generalizability, interpretability, and seamless clinical integration. Overcoming these obstacles requires a concerted effort focusing on rigorous prospective validation, the development of robust and explainable AI models, and careful integration into existing clinical workflows. By fostering interdisciplinary collaboration among AI researchers, clinicians, ethicists, and regulators, and by prioritizing demonstrable clinical impact over mere algorithmic performance, AI holds immense promise. It can fundamentally improve patient outcomes, optimize resource utilization, and ultimately transform the landscape of ARDS care, paving the way for a new paradigm of human-AI partnership in critical care.

## References

[1] Koulouras V Papathanakos G Papathanasiou A Nakos G. Efficacy of prone position in acute respiratory distress syndrome patients: a pathophysiology-based review. World J Crit Care Med. (2016) 5:121. doi: 10.5492/wjccm.v5.i2.121, PMID: 27152255 PMC4848155

[2] Wu HP Leu SW Lin SW Hung CY Chen NH Hu HC. Role of changes in driving pressure and mechanical power in predicting mortality in patients with acute respiratory distress syndrome. Diagnostics. (2023) 13:1226. doi: 10.3390/diagnostics13071226, PMID: 37046444 PMC10093066

[3] Maddali MV Churpek M Pham T Rezoagli E Zhuo H Zhao W. Validation and utility of ARDS subphenotypes identified by machine-learning models using clinical data: an observational, multicohort, retrospective analysis. Lancet Respir Med. (2022) 10:367–77. doi: 10.1016/S2213-2600(21)00461-6, PMID: 35026177 PMC8976729

[4] Bai Y Xia J Huang X Chen S Zhan Q. Using machine learning for the early prediction of sepsis-associated ARDS in the ICU and identification of clinical phenotypes with differential responses to treatment. Front Physiol. (2022) 13:1050849. doi: 10.3389/fphys.2022.1050849 36579020 PMC9791185

[5] Yang J Zeng S Cui S Zheng J Wang H. Predictive modeling of acute respiratory distress syndrome using machine learning: systematic review and meta-analysis. J Med Internet Res. (2025) 27:e66615. doi: 10.2196/66615, PMID: 40359510 PMC12117268

[6] Silversides JA Ferguson ND. Clinical review: acute respiratory distress syndrome - clinical ventilator management and adjunct therapy. Crit Care. (2013) 17:225. doi: 10.1186/cc11867, PMID: 23672857 PMC3672489

[7] Sinha P Churpek MM Calfee CS. Machine learning classifier models can identify acute respiratory distress syndrome phenotypes using readily available clinical data. Am J Respir Crit Care Med. (2020) 202:996–1004. doi: 10.1164/rccm.202002-0347OC, PMID: 32551817 PMC7528785

[8] Rehm GB Cortés-Puch I Kuhn BT Nguyen J Fazio SA Johnson MA. Use of machine learning to screen for acute respiratory distress syndrome using raw ventilator waveform data. Crit Care Explor. (2021) 3:e0313. doi: 10.1097/CCE.0000000000000313, PMID: 33458681 PMC7803688

[9] Reamaroon N Sjoding MW Gryak J Athey BD Najarian K Derksen H. Automated detection of acute respiratory distress syndrome from chest X-rays using directionality measure and deep learning features. Comput Biol Med. (2021) 134:104463. doi: 10.1016/j.compbiomed.2021.104463, PMID: 33993014 PMC9169678

[10] Zhou Y Feng J Mei S Tang R Xing S Qin S. A deep learning model for predicting COVID-19 ARDS in critically ill patients. Front Med. (2023) 10:1221711. doi: 10.3389/fmed.2023.1221711, PMID: 37564041 PMC10411521

[11] Tan R Ge C Li Z Yan Y Guo H Song W. Early prediction of mortality risk in acute respiratory distress syndrome: systematic review and meta-analysis. J Med Internet Res. (2025) 27:e70537. doi: 10.2196/70537, PMID: 40392588 PMC12134695

[12] Deng Y Li S Li J Tao X Li Y You C. Enhancing mortality prediction in intensive care units: improving APACHE II, SOFA, and SAPS II scoring systems using long short-term memory. Intern Emerg Med. (2025) 5:896. doi: 10.1007/s11739-025-03896-5, PMID: 40325281

[13] Hannon DM Syed JDA McNicholas B Madden M Laffey JG. The development of a C5.0 machine learning model in a limited data set to predict early mortality in patients with ARDS undergoing an initial session of prone positioning. Intensive Care Med Exp. (2024) 12:103. doi: 10.1186/s40635-024-00682-z 39540987 PMC11564488

[14] Li W Zhou H Zou Y. An interpretable machine learning model for predicting mortality risk in adult ICU patients with acute respiratory distress syndrome. Front Med. (2025) 12:1580345. doi: 10.3389/fmed.2025.1580345 PMC12061690 40351465

[15] Calfee CS Delucchi K Parsons PE Thompson BT Ware LB Matthay MA. Subphenotypes in acute respiratory distress syndrome: latent class analysis of data from two randomised controlled trials. Lancet Respir Med. (2014) 2:611–20. doi: 10.1016/S2213-2600(14)70097-9, PMID: 24853585 PMC4154544

[16] Matthay MA Arabi YM Siegel ER Ware LB Bos LDJ Sinha P. Phenotypes and personalized medicine in the acute respiratory distress syndrome. Intensive Care Med. (2020) 46:2136–52. doi: 10.1007/s00134-020-06296-9, PMID: 33206201 PMC7673253

[17] Calfee CS Delucchi KL Sinha P Matthay MA Hackett J Shankar-Hari M. Latent class analysis of ARDS subphenotypes: a secondary analysis of the statins for acutely injured lungs from sepsis (SAILS) study. Lancet Respir Med. (2018) 6:691–8. doi: 10.1016/S2213-2600(18)30177-2, PMID: 30291376 PMC6317524

[18] Rashid M Ramakrishnan M Chandran VP Nandish S Nair S Shanbhag V. Artificial intelligence in acute respiratory distress syndrome: a systematic review. Artif Intell Med. (2022) 131:102361. doi: 10.1016/j.artmed.2022.102361, PMID: 36100348

[19] Famous KR Delucchi K Ware LB Kangelaris KN Liu KD Thompson BT. Acute respiratory distress syndrome subphenotypes respond differently to randomized fluid management strategy. Am J Respir Crit Care Med. (2017) 195:331–8. doi: 10.1164/rccm.201603-0645OC, PMID: 27513822 PMC5328179

[20] Shaver CM Bastarache JA. Clinical and biological heterogeneity in acute respiratory distress syndrome. Clin Chest Med. (2014) 35:639–53. doi: 10.1016/j.ccm.2014.08.004, PMID: 25453415 PMC4254695

[21] Meza-Fuentes G Delgado I Barbé M Sánchez-Barraza I Retamal MA López R. Machine learning-based identification of efficient and restrictive physiological subphenotypes in acute respiratory distress syndrome. Intensive Care Med Exp. (2025) 13:29. doi: 10.1186/s40635-025-00737-9 40024962 PMC11872963

[22] Viderman D Ayazbay A Kalzhan B Bayakhmetova S Tungushpayev M Abdildin Y. Artificial intelligence in the management of patients with respiratory failure requiring mechanical ventilation: a scoping review. J Clin Med. (2024) 13:7535. doi: 10.3390/jcm13247535, PMID: 39768462 PMC11728182

[23] Beitler JR Di Gennaro E van Amstel RBE Beenen LFM Grasso S. Latent class analysis of imaging and clinical respiratory parameters from patients with COVID-19-related ARDS identifies recruitment subphenotypes. Lancet Respir Med. (2021) 9:1401–12. doi: 10.1186/s13054-022-04251-2 PMC9700924 36434629

[24] Nishikimi M Ohshimo S Bellani G Fukumoto W Anzai T Liu K. Identification of novel sub-phenotypes of severe ARDS requiring ECMO using latent class analysis. Crit Care. (2024) 28:343. doi: 10.1186/s13054-024-05143-3, PMID: 39449081 PMC11515347

[25] Lin M Xu F Sun J Song J Shen Y Lu S. Integrative multi-omics analysis unravels the host response landscape and reveals a serum protein panel for early prognosis prediction for ARDS. Crit Care. (2024) 28:213. doi: 10.1186/s13054-024-05000-3, PMID: 38956604 PMC11218270

[26] Cui H Huang X. Multi-omics integration reveals YWHAE as a key mediator of ferroptosis in ARDS. Funct Integr Genomics. (2025) 25:94. doi: 10.1007/s10142-025-01603-3 40261442

[27] Overmyer KA Shishkova E Miller IJ Balnis J Bernstein MN Peters-Clarke TM. Large-scale multi-omic analysis of COVID-19 severity. Cell Syst. (2021) 12:23–40.e7. doi: 10.1016/j.cels.2020.10.003, PMID: 33096026 PMC7543711

[28] Sella N Pettenuzzo T Zarantonello F Andreatta G De Cassai A Schiavolin C. Electrical impedance tomography: a compass for the safe route to optimal PEEP. Respir Med. (2021) 187:106555. doi: 10.1016/j.rmed.2021.106555, PMID: 34352563

[29] Händel C Frerichs I Weiler N Bergh B. Prediction and simulation of PEEP setting effects with machine learning models. Med Intensiva. (2024) 48:191–9. doi: 10.1016/j.medin.2023.09.009 38135579

[30] Chiew YS Chase JG Shaw GM Sundaresan A Desaive T. Model-based PEEP optimisation in mechanical ventilation. Biomed Eng Online. (2011) 10:111. doi: 10.1186/1475-925X-10-111 22196749 PMC3339371

[31] Rietveld TP Van Der Ster BJP Schoe A Endeman H Balakirev A Kozlova D. Let’s get in sync: current standing and future of AI-based detection of patient-ventilator asynchrony. Intensive Care Med Exp. (2025) 13:39. doi: 10.1186/s40635-025-00746-8, PMID: 40119215 PMC11928342

[32] Tlimat A Fowler C Safadi S Johnson RB Bodduluri S Morris P. Artificial intelligence for the detection of patient–ventilator asynchrony. Respir Care. (2025) 70:583–92. doi: 10.1089/respcare.12540, PMID: 40178919 PMC12369843

[33] Stell A Caparo EC Wang Z Wang C Berlowitz D Howard M. (2024) Identification of patient ventilator asynchrony in physiological data through integrating machine-learning. doi: 10.5220/0012366700003657

[34] Gattinoni L Tonetti T Cressoni M Cadringher P Herrmann P Moerer O. Ventilator-related causes of lung injury: the mechanical power. Intensive Care Med. (2016) 42:1567–75. doi: 10.1007/s00134-016-4505-2, PMID: 27620287

[35] Kim TW Chung CR Nam M Ko RE Suh GY. Associations of mechanical power, ventilatory ratio, and other respiratory indices with mortality in patients with acute respiratory distress syndrome undergoing pressure-controlled mechanical ventilation. Front Med. (2025) 12:1553672. doi: 10.3389/fmed.2025.1553672 PMC12006839 40255591

[36] Alkhalifah AS Rumindo K Brincat E Blanchard F Helleberg J Clarke D. Optimizing mechanical ventilation: personalizing mechanical power to reduce ICU mortality - a retrospective cohort study. PLoS One. 20:e0318018. doi: 10.1371/journal.pone.0318018 PMC11825045 39946423

[37] Chang KW Leu SW Hu HC Chan MC Liang SJ Yang KY. The mechanical power in patients with acute respiratory distress syndrome undergoing prone positioning can predict mortality. Diagnostics. (2025) 15:158. doi: 10.3390/diagnostics15020158, PMID: 39857042 PMC11763726

[38] Fosset M Von Wedel D Redaelli S Talmor D Molinari N Josse J. Subphenotyping prone position responders with machine learning. Crit Care. (2025) 29:116. doi: 10.1186/s13054-025-05340-8 40087660 PMC11909901

[39] Trieu M Qadir N. Adjunctive therapies in acute respiratory distress syndrome. Crit Care Clin. (2024) 40:329–51. doi: 10.1016/j.ccc.2023.12.004, PMID: 38432699

[40] Zhu D Xue B Shah N Payne PRO Lu C Said AS. Multi-modal prediction of extracorporeal support—a resource intensive therapy, utilizing a large national database. JAMIA Open. (2024) 8:ooae158. doi: 10.1093/jamiaopen/ooae158 PMC11702361 39764170

[41] Stephens AF Šeman M Diehl A Pilcher D Barbaro RP Brodie D. ECMO PAL: using deep neural networks for survival prediction in venoarterial extracorporeal membrane oxygenation. Intensive Care Med. (2023) 49:1090–9. doi: 10.1007/s00134-023-07157-x, PMID: 37548758 PMC10499722

[42] Igarashi Y Ogawa K Nishimura K Osawa S Ohwada H Yokobori S. Machine learning for predicting successful extubation in patients receiving mechanical ventilation. Front Med. (2022) 9:961252. doi: 10.3389/fmed.2022.961252, PMID: 36035403 PMC9403066

[43] Xu Y Xue J Deng Y Tu L Ding Y Zhang Y. Advances in machine learning for mechanically ventilated patients. Int J Gen Med. (2025) 18:3301–11. doi: 10.2147/IJGM.S515170 40568522 PMC12191144

[44] Huang KY Hsu YL Chen HC Horng MH Chung CL Lin CH. Developing a machine-learning model for real-time prediction of successful extubation in mechanically ventilated patients using time-series ventilator-derived parameters. Front Med. (2023) 10:1167445. doi: 10.3389/fmed.2023.1167445 PMC10203709 37228399

[45] Lin YH Chang TC Liu CF Lai CC Chen CM Chou W. The intervention of artificial intelligence to improve the weaning outcomes of patients with mechanical ventilation: practical applications in the medical intensive care unit and the COVID-19 intensive care unit: a retrospective study. Medicine. (2024) 103:e37500. doi: 10.1097/MD.0000000000037500, PMID: 38518051 PMC10956977

[46] Liao KM Ko SC Liu CF Cheng KC Chen CM Sung MI. Development of an interactive AI system for the optimal timing prediction of successful weaning from mechanical ventilation for patients in respiratory care centers. Diagnostics. (2022) 12:975. doi: 10.3390/diagnostics12040975, PMID: 35454023 PMC9030191

[47] Liu CF Hung CM Ko SC Cheng KC Chao CM Sung MI. An artificial intelligence system to predict the optimal timing for mechanical ventilation weaning for intensive care unit patients: a two-stage prediction approach. Front Med. (2022) 9:935366. doi: 10.3389/fmed.2022.935366, PMID: 36465940 PMC9715756

[48] Kamya P Ozerov IV Pun FW Tretina K Fokina T Chen S. PandaOmics: an AI-driven platform for therapeutic target and biomarker discovery. J Chem Inf Model. (2024) 64:3961–9. doi: 10.1021/acs.jcim.3c01619, PMID: 38404138 PMC11134400

[49] Johnson R Li MM Noori A Queen O Zitnik M. Graph artificial intelligence in medicine. Annu Rev Biomed Data Sci. (2024) 7:345–68. doi: 10.1146/annurev-biodatasci-110723-024625, PMID: 38749465 PMC11344018

[50] Chen X Harhay MO Tong G Li F. A bayesian machine learning approach for estimating heterogeneous survivor causal effects: applications to a critical care trial. Ann Appl Stat. (2024) 18:350–74. doi: 10.1214/23-aoas1792, PMID: 38455841 PMC10919396

[51] Ervin JN Rentes VC Dibble ER Sjoding MW Iwashyna TJ Hough CL. Evidence-based practices for acute respiratory failure and acute respiratory distress syndrome. Chest. (2020) 158:2381–93. doi: 10.1016/j.chest.2020.06.080 32682771 PMC7768938

[52] Rilinger J Zotzmann V Bemtgen X Schumacher C Biever PM Duerschmied D. Prone positioning in severe ARDS requiring extracorporeal membrane oxygenation. Crit Care. (2020) 24:397. doi: 10.4187/respcare.05752 32641155 PMC7341706

[53] Bos LD Sjoding M Sinha P Bhavani SV Lyons PG Bewley AF. Longitudinal latent class analysis of ventilation parameters in COVID-19 related acute respiratory distress syndrome identifies dynamic subphenotypes. Lancet Respir Med. (2021) 9:1121–30. doi: 10.1016/S2213-2600(21)00365-934048716

[54] Rodriguez PO Tiribelli N Gogniat E Plotnikow GA Fredes S. Automatic detection of reverse-triggering related asynchronies during mechanical ventilation in ARDS patients using flow and pressure signals. J Clin Monit Comput. (2020) 34:1239–46. doi: 10.1007/s10877-019-00444-3, PMID: 31853811

